# Changes in substance use during outpatient treatment for substance use disorders: a prospective Norwegian cohort study from 2016 to 2020

**DOI:** 10.1186/s13011-021-00403-9

**Published:** 2021-09-15

**Authors:** Jørn Henrik Vold, Fatemeh Chalabianloo, Christer F. Aas, Else-Marie Løberg, Kjell Arne Johansson, Lars Thore Fadnes

**Affiliations:** 1 Department of Addiction Medicine, Haukeland University Hospital, Bergen, Norway; 2 Department of Global Public Health and Primary Care, University of Bergen, Bergen, Norway; 3 Department of Psychiatry, Haukeland University Hospital, Bergen, Norway; 4 Department of Clinical Psychology, University of Bergen, Bergen, Norway

**Keywords:** Substance-related disorders, Substance use, Inpatient detoxification, Opiate substitution treatment, Comorbidities, Illicit drugs, Low-threshold health services

## Abstract

**Background:**

Continuous use of amphetamines, alcohol, benzodiazepines, cannabis, cocaine, or opioids contributes to health impairments, increased morbidity, and overdose deaths among patients with substance use disorders (SUDs). This study evaluates the impact of inpatient detoxification, injecting substance use, age, and gender on substance use over time among patients undergoing outpatient SUD treatment.

**Methods:**

We used data from a cohort of SUD patients in Norway obtained from health assessments of self-reported substance use and sociodemographic and clinical factors. A total of 881 substance use measurements, including substances and frequency of use, were assessed for 708 SUD patients in 2016–2020. Of those, 171 patients provided two or more substance use measurements. The total substance use was calculated, creating a substance use severity index (SUSI), ranging from zero (no use) to one (daily use of all substances). We defined *baseline* as the first substance use measurement when the measurements were listed chronologically. *Time* was defined as years from baseline. We used a linear mixed model to analyze the SUSI at baseline and over time, and its associations with inpatient detoxification, injecting substance use, gender, and age, presented with coefficients and 95% confidence intervals (CI).

**Results:**

No longitudinal changes in the SUSI were found compared with baseline (change in SUSI (cSUSI): 0.04, 95% CI: − 0.05;0.13, *p* = 0.397). Likewise, “inpatient detoxification” was not associated with changes in the SUSI compared with “no inpatient detoxification” (cSUSI: 0.00, 95% CI: − 0.04;0.04, *p* = 0.952). However, injecting substances were associated with a higher SUSI than not injecting substances at baseline (difference in SUSI: 0.19, 95% CI: 0.16;0.21, *p* = < 0.001), and starting to inject substances was associated with increasing SUSI over time compared with not starting to inject substances (cSUSI: 0.11, 95% CI: 0.07;0.15, *p* = < 0.001). Gender was not significantly associated with changes in the SUSI (cSUSI: − 0.04, 95% CI: − 0.07;0.00, *p* = 0.052), while patients over 60 years of age had a lower SUSI than those under the age of 30 at baseline (difference in SUSI: − 0.08, 95% CI: − 0.14;− 0.01, *p* = 0.018), with no change over time (cSUSI: − 0.05, 95% CI: − 0.16;0.05, *p* = 0.297).

**Conclusion:**

The present study demonstrates that inpatient detoxification was not associated with substance use changes over time for patients undergoing outpatient SUD treatment. Otherwise, injecting substance use was a particular risk factor for a high level of substance use. Future research needs to evaluate the impact of other treatment approaches on substance use, ideally in randomized controlled trials.

**Supplementary Information:**

The online version contains supplementary material available at 10.1186/s13011-021-00403-9.

## Background

More than half of patients with substance use disorders (SUDs) use addictive substances continuously while enrolled in SUD treatment [1, 2]. Continuous substance use diminishes treatment effects and is associated with health adversities, morbidity, and overdose deaths [3, 4]. In 2020, the European Union presented a substance strategy for 2021–2025 to reduce the substance demand, dependence, and supply of substances and address substance-related health and social harms by 2025 [5]. As part of this ambitious strategy, increased opioid agonist therapy (OAT) coverage is an essential approach for preventing injecting opioid use [6], reducing premature mortality [7], and limiting illegal opioid consumption by SUD patients with severe opioid dependence [8, 9]. However, the extent to which inpatient detoxification, injecting substance use, age, and gender impact total substance use among patients undergoing outpatient SUD treatment in OAT or municipal treatment centers remains unclear [10, 11].

Patients engaged in continuous substance use suffer from multiple disease burdens, and many have physical and mental comorbidities and socioeconomic difficulties [4]. Mental comorbidities – such as personality disorders and psychotic and affective disorders – are common [12–14]. Additionally, there is a high prevalence of hepatitis C virus (HCV) and human immunodeficiency virus (HIV) infections, endocarditis, and bacterial abscesses, related to injecting substance use [15–19]. Chaotic life situations are present in many cases, involving unstable housing situations, unemployment, and disrupted family and social relationships. Additionally, continuous substance use constitutes a particular risk of fatal and non-fatal overdoses [18], typically when opioids are combined with other sedatives or alcohol [20]. The European Monitoring Centre of Drug and Drug Addiction has estimated a wide variance in the percentage of OAT patients who use substances other than opioids (11–70%) [4]. For Europeans engaged in harmful opioid use, benzodiazepine consumption has been reported to range from 12 to 85% [21, 22].

Inpatient detoxification may be a step toward rehabilitation and recovery from continuous substance use for patients undergoing outpatient SUD treatment. Inpatient detoxification usually involves medical and psychosocial follow-ups targeting a range of physical and mental substance withdrawal symptoms, such as nausea, tremors, sweating, irritability, insomnia, hallucinations, seizures, and anxiety [23]. However, studies have shown that substance use relapse is common, and few detoxified patients remain substance abstinent in the long term [24–27].

Thus, the present study’s objectives are to evaluate continuous substance use in terms of the type and amount of consumed substances, including alcohol, amphetamines, benzodiazepines, cannabis, cocaine, and opioids, over time among Norwegian patients with substance use disorder (SUD) receiving outpatient SUD treatment in opioid agonist therapy (OAT) or municipal treatment centers using a substance use severity index (SUSI). In addition, we aim to assess inpatient detoxification and injecting substance use and their associations with changes in substance use over time. More specifically, we will:

1. calculate the substance use at baseline and assess changes over time.
2. evaluate the impact of inpatient detoxification, injecting substance use, age, and gender on the substance use at baseline and over time.

## Methods

### Data source

We used data from a cohort nested to the INTRO-HCV study in Bergen and Stavanger, Norway [28]. Data were collected from May 2016 to July 2020, and patients were recruited from OAT outpatient clinics in Bergen and Stavanger and from municipal outpatient SUD treatment centers in the Bergen Municipality.

### Data collections

All included patients were assessed yearly regarding their physical and mental health status, current substance use, and sociodemographic and clinical data. The data were collected in a health register using electronic data collection software (Checkware) under the supervision of research nurses. All of the clinical data, including education level, inpatient detoxification, severe infectious diseases (HCV, hepatitis B virus, and HIV infections), and substance use were collected from the electronic medical record.

### Study sample

We included 881 self-reported substance use measurements from 708 patients with SUDs in the study period. Of those, 171 patients provided two or more substance use measurements, providing 346 repeated measurements. The median time interval between the first health assessments (baseline), and any subsequent assessments of the same patients, including substance use measurements, was 16 months (interquartile range (IQR): 13–20).

### Measuring substance use

We measured substance use during the past 12 months prior to the assessments using an objective substance use scale for each substance class, including alcohol, benzodiazepines, cannabis, opioids (opioids received in OAT were not included), and stimulants (amphetamines or cocaine). The scale ranges from zero to five points, where zero represents “never,” one represents “less than one day per month,” two represents “one to three days per month,” three represents “one to three days per week,” four represents “more than three days per week,” and five represents “daily” use of a substance. The substance scores (0–5) were handled separately for each substance class and were additionally summarized as a sum score ranging from zero to 25 points (Additional File 1). Furthermore, the scores were customized into a continuous SUSI ranging from zero (no use) to one (daily use) by dividing the total substance sum score by five (for individual substance class) or 25 (for all substance classes) in order to simplify the scales and make it easier to interpret the results of different substance classes. The data collection software only allowed valid responses to each substance and prompted empty questions before submission to minimize missing data.

### Definition of study variables

We defined the baseline for patients as the first health assessment that included a substance use measurement when we listed the health assessments chronologically. The age variable was classified into five groups: 18–30, 30–40, 40–50, 50–60, and ≥ 60 years of age. We defined “injecting substance use” as having injected at least once at any time during the past 6 months prior to the health assessment. Additionally, we defined inpatient detoxification as being hospitalized for detoxification at least once between baseline and the last substance use measurement. By detoxification, we mean detoxification of illegal substances and alcohol without tapering off or discontinuing OAT opioids. The duration of inpatient detoxification was not considered. Furthermore, we assessed the extent of hepatitis B and C virus and HIV infections as markers of the study populations’ comorbidities by drawing blood samples, including hepatitis B surface antigens, HIV antigens/antibodies, and HCV polymerase chain reaction during health assessments. Having chronic infectious diseases was defined as detecting HCV RNA by polymerase chain reaction, hepatitis B virus surface antigens, or HIV antigen/antibodies in the blood samples. Blood samples were analyzed at the Department of Laboratory Medicine, Haukeland University Hospital, Bergen, Norway, and the Department of Medical Biochemistry and Microbiology, Stavanger University Hospital, Stavanger, Norway (accredited by ISO standard 15189).

### Statistical analyses

We used Stata/SE 17.0 (StataCorp, TX, USA) for the descriptive and linear mixed model analyses. Sankeymatic (sankeymatic.com) was used to generate a Sankey diagram for graphical presentation of the change in substance use over time. The threshold for statistical significance was set to *P* < 0.05 for all analyses unless otherwise stated. In all analyses, we defined *time* as the number of years from baseline.

Linear mixed model analyses were used to investigate whether inpatient detoxification, injecting substance use, age, and gender affected the SUSI at baseline and the extent to which they influenced any changes in the SUSI from baseline to the following health assessments. The predictors were handled both as constant baseline variables and as time-varying variables, with the SUSI as the outcome variable. The longitudinal analysis calculated the mean change in the SUSI (cSUSI) within the predictor groups. This was defined as the mean change per year in the SUSI from baseline within these groups, after subtracting any corresponding mean change in the SUSI from baseline within the comparator groups. We specified the linear mixed models as random intercept fixed slope regression models. The estimator was set to Restricted Maximum Likelihood. The full information maximum likelihood ensured that all available substance use measurements were used.

### Ethics approval and consent to participate

The study was reviewed and approved by the Regional Ethical Committee for Health Research West, Norway (REK Vest 2017/51). Each patient provided written informed consent prior to enrolling in the study.

## Results

### Patients’ characteristics at baseline

Seventy-three percent of the study sample were males, and the mean age was 43 years (standard deviation (SD): 11 years) at baseline (Table 1). Seventy-eight percent were recruited from the OAT outpatient clinics, and the remaining samples were from the municipal SUD treatment centers. Six percent had not completed primary school, and 44% had primary school as their highest education level. Fifty-four percent injected substances during the past 6 months leading up to the substance use measurement, and 20% were admitted to at least one inpatient detoxification in the time between baseline and the last health assessment. Ninety-six percent had consumed at least one substance during the past 12 months before conducting the first health assessment.

**Table 1:** Basic characteristics at baseline for all patients and for patients with more than one substance use measurement (numbers (n) and percentages (%))

	All patients (*N* = 708)	Patients with > 1 substance use measurement (*N* = 171)
*Age (years), n (%)*		
18–30	84 (12)	11 (6)
30–40	204 (29)	43 (25)
40–50	215 (30)	57 (33)
50–60	164 (23)	47 (27)
≥ 60	41 (6)	13 (8)
Mean (SD)	43 (11)	46 (10)
Gender, *n (%)*		
Male	514 (73)	130 (76)
Female	194 (27)	41 (24)
Highest education level, *n (%)*		
Not completed primary school	39 (6)	11 (6)
Completed primary school (9 years)	306 (44)	77 (45)
Completed high school (12 years)	277 (40)	66 (39)
≤ 3 years of college or university	58 (8)	< 15 (< 10)
> 3 years of college or university	14 (2)	< 15 (< 10)
*Injected substances during the past 6 months, n (%)*	384 (54)	82 (48)
*Unstable housing situation during the past 30 days*^a^*, n (%)*	86 (12)	5 (4)
*Substance use during the past 12 months*^b^, *n (%)*		
Alcohol	513 (72)	113 (66)
Benzodiazepines	489 (69)	126 (74)
Cannabis	537 (76)	133 (78)
Opioids	344 (49)	75 (44)
Stimulants (amphetamines and cocaine)	451 (64)	103 (60)
*Inpatient detoxification, n (%)*	–	35 (20)
*Received OAT, n (%)*	553 (78)	166 (97)
*Received municipality care, n (%)*	155 (22)	5 (3)
*Comorbidities, n (%)*		
Hepatitis C virus infection	349 (60)	89 (57)
Hepatitis B virus infection	5 (< 1)	< 5 (< 5)
Human immunodeficiency virus infection	< 5 (< 5)	< 5 (< 5)

*SD* Standard deviation

^a^An unstable housing situation was defined as living in a homeless shelter or with family or friends at any time during the past 30 days. Having owned or rented housing situation or being imprisoned were defined as a stable housing situation

^b^The number of patients who have used substances at least once during the past 12 months

### Substance use severity index at baseline and over time

**Fig. 1:**
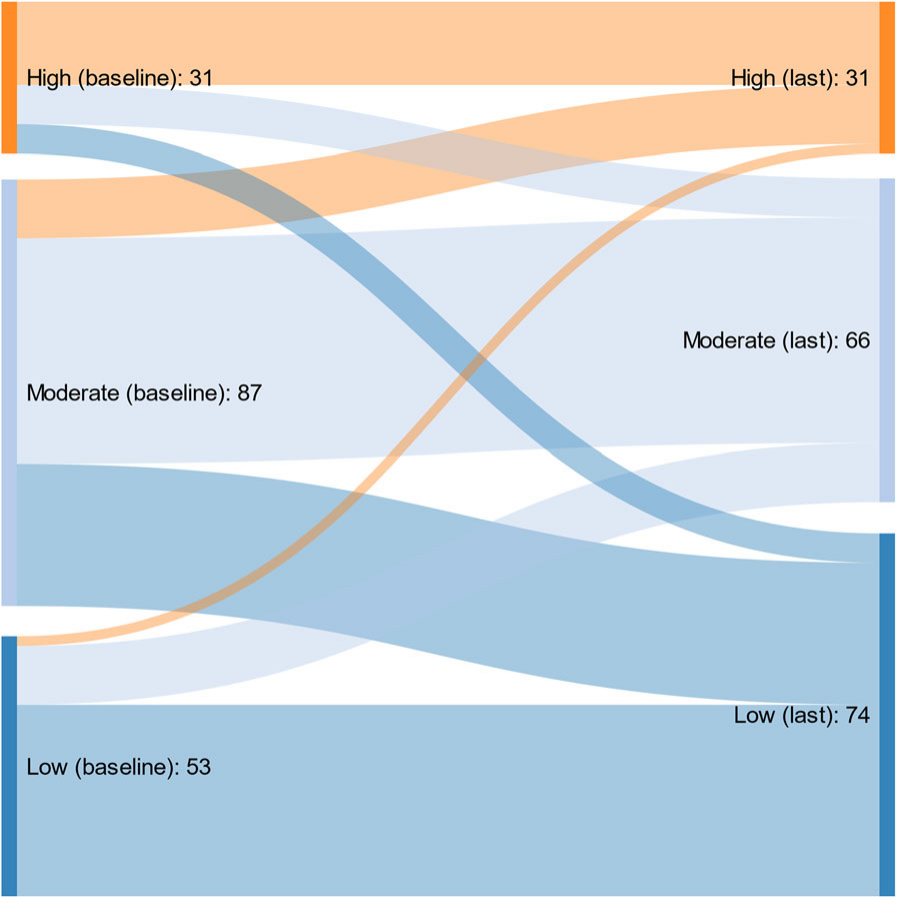
Patients’ Substance Use Severity Index (SUSI) changes from baseline to the last substance use measurement (*N* = 171). Legends: The figure displays the SUSI changes from baseline (left (baseline)) to the last substance use measurement (right (last)) for patients with at least two substance use measurements. Patients were divided into three SUSI levels at baseline and the last substance use measurement: low (SUSI < 0.2 (dark blue)), moderate (SUSI 0.2–0.4 (light blue)), and high (SUSI > 0.4 (orange)) substance use. The SUSI ranges from 0 to 1, where 0 indicates no substance use and 1 indicates daily substance use for all substances (cannabis, amphetamines, cocaine, opioids, benzodiazepines, and alcohol)

The mean SUSI for all substances was 0.35 (SD: 0.20) at baseline (Table 2). Cannabis was the most frequently used substance (mean score: 0.50 (0.38)), followed by benzodiazepines (0.40 (0.37)), stimulants (0.33 (0.34)), alcohol (0.31 (0.29)), and opioids (0.22 (0.31)). Furthermore, the SUSI was substantially unchanged from the first to the last substance use measurement, with a tendency toward a slight reduction (Fig. 1).

**Table 2:** The Substance Use Severity Index (SUSI) (mean (SD)) at baseline and follow-up

	*Baseline*^a^ *(N = 708)*	*Baseline*^b^ *(N = 171)*	*Follow-up*^c^ *(N = 171)*
Alcohol	0.31 (0.29)	0.29 (0.29)	0.28 (0.27)
Cannabis	0.50 (0.38)	0.51 (0.38)	0.50 (0.40)
Benzodiazepines	0.40 (0.37)	0.42 (0.36)	0.39 (0.34)
Opioids	0.22 (0.31)	0.21 (0.31)	0.14 (0.23)
Stimulants (amphetamines and cocaine)	0.33 (0.34)	0.28 (0.31)	0.25 (0.31)
All substances	0.35 (0.20)	0.34 (0.18)	0.31 (0.18)

Each substance class and the total substance use (“All substances”) are customized into the SUSI, ranging from 0 to 1, where 0 indicates no use and 1 indicates daily use of all substances (alcohol, cannabis, benzodiazepines, opioids, and stimulants). SD: Standard Deviation

^a^The SUSI for all included patients at baseline

^b^The SUSI for patients with two or more substance use measurements at baseline

^c^The SUSI for patients with two or more substance use measurements on the last substance use assessment during the study period

### The associations between the substance use severity index (all substances included), and inpatient detoxification, injecting substance use, age, and gender at baseline

At baseline, no difference in the SUSI was found between patients who were admitted to inpatient detoxification and those who were not admitted to inpatient detoxification (difference in SUSI with inpatient detoxification and without inpatient detoxification: − 0.02, 95% confidence interval (CI): − 0.03;0.08, *p* = 0.422) (Table 3). Patients who injected substances had a higher SUSI than those who did not inject substances (difference in SUSI of injecting substances and not injecting substances: 0.19, 95% CI: 0.16;0.21, *p* = < 0.001), while patients over 60 years of age were associated with a lower SUSI than those under the age of 30 (difference in SUSI of “over 60 years of age” and “under 30 years of age”: − 0.08, 95% CI: − 0.14;-0.01, *p* = 0.018). Substantially similar results were found when only including patients with two or more SUSI measurements in the analyses (Additional File 2).

**Table 3:** Adjusted linear mixed model for the Substance Use Severity Index (SUSI)^a^ (*N* = 706)

	Effect estimates	
	Coefficients (95% CI)	*p*-value
Substance Use Severity Index (β_0_)	0.29 (0.24;0.33)	< 0.001
Time trend	0.04 (−0.05;0.13)	0.397
*Baseline*		
Female	-0.01 (− 0.04;0.01)	0.336
Years of age:		
< 30	0.00 (ref.)	
30–40	−0.04 (− 0.08;0.01)	0.091
40–50	−0.03 (− 0.07;0.01)	0.190
50–60	− 0.04 (− 0.09;0.00)	0.055
≥ 60	−0.08 (− 0.14;-0.01)	0.018
Injecting substance use	0.19 (0.16;0.21)	< 0.001
Inpatient detoxification^b^	0.02 (−0.03;0.08)	0.422
*Predictors remain constant from baseline*^c^		
Injecting substance use	0.01 (−0.02;0.05)	0.542
Female	−0.04 (− 0.07;0.00)	0.052
Years of age:		
< 30	0.00 (ref.)	
30–40	−0.07 (− 0.16;0.01)	0.098
40–50	−0.05 (− 0.13;0.04)	0.293
50–60	− 0.05 (− 0.14;0.03)	0.229
≥ 60	−0.05 (− 0.16;0.05)	0.297
*Time-varying predictors from baseline*^d^		
Inpatient detoxification	0.00 (−0.04;0.04)	0.952
Starting to inject substances	0.11 (0.07;0.15)	< 0.001

The table displays a linear mixed model analysis (Restricted Maximum Likelihood regression) evaluating the impact of inpatient intoxication, injecting substance use, age, and gender on the SUSI at baseline and from baseline (over time) among patients undergoing outpatient SUD treatment

*CI* Confidence Interval, *OAT* Opioid Agonist Therapy, *cSUSI* Mean Change in Substance Use Severity Index, *SUD* Substance Use Disorder

^a^The SUSI is a continuous variable ranging from 0 to 1, where 0 indicates no substance use and 1 indicates daily substance use for all substances (cannabis, amphetamines, cocaine, opioids, benzodiazepines, and alcohol)

^b^The value shows the difference in adjusted SUSI between “going to inpatient detoxification” and “not going to inpatient detoxification” at baseline (before detoxification)

^c^Interpretation: the cSUSI per year from baseline of a constant predictor and its comparator (e.g., the cSUSI of “ongoing injecting substance use” (constant predictor) and “no ongoing inject substance use” (constant comparator) per year from baseline)

^d^Interpretation: the cSUSI of a time-varying predictor and its time-varying comparator per year from baseline (e.g., the cSUSI per year of “going to inpatient detoxification” and “not going to inpatient detoxification” from baseline)

### The associations between the substance use severity index (all substances included), and inpatient detoxification, injecting substance use, age, and gender per year from baseline

No significant longitudinal changes in the SUSI were found in the entire population (cSUSI from baseline per year: 0.04, 95% CI: − 0.05;0.13, *p* = 0.397). Likewise, admitting to inpatient detoxification was not associated with longitudinal changes in the SUSI compared with not admitting to inpatient detoxification (cSUSI with inpatient detoxification [predictor] and without inpatient detoxification [comparator]: 0.00, 95% CI: − 0.04;0.04, *p* = 0.952). However, gender was not significantly associated with changes in the SUSI (cSUSI of females [predictor] and males [comparator]: − 0.04, 95% CI: − 0.07;0.00, *p* = 0.052), while an increasing SUSI over time was associated with patients who started to inject substances compared with those who did not start to inject from baseline (cSUSI of starting to inject substance [predictor] and no starting to inject substance [comparator]: 0.11, 95% CI: 0.07;0.15, *p* = < 0.001).

## Discussion

The present study revealed considerable substance use among patients undergoing outpatient SUD treatment. Cannabis was the most common substance used, followed by benzodiazepines, stimulants, alcohol, and opioids. Furthermore, patients admitted to inpatient detoxification were not associated with changes in substance use from baseline compared with those who were not admitted to inpatient detoxification. However, patients over 60 years of age had lower substance use than patients under 30 years of age at baseline, with no change over time. Higher substance use was found among patients who injected substances at baseline and those who started to inject substances. Otherwise, gender was not significantly associated with changes in substance use over time.

The substance use levels were high among SUD patients undergoing OAT or municipal SUD treatment, with levels exceeding the prevalence estimates of benzodiazepine and stimulant use in the national data on the OAT population in Norway [29]. Additionally, the benzodiazepine prevalence estimate was similar to the higher estimates in European countries among harmful opioid users, ranging from 45 to 70% [21]. The higher availability of illegal substances and consequently higher number of SUD patients in bigger cities than in other regions could be a possible reason for these findings.

The findings indicated that there was no additional benefit of inpatient detoxification, indicating that reduced substance use does not seem to be the outcome of inpatient detoxification treatment. However, other indirect effects of detoxification could occur. Most SUD patients were marginalized substance users with different health and social problems, in addition to the current SUDs, which needed to be addressed during inpatient detoxification [3]. Thus, inpatient detoxification should be one of several approaches in a comprehensive treatment course for these patients. Nevertheless, reducing substance use may be difficult, and substance use relapse may even be expected for many of these patients. A previous study found that younger patients, patients with a psychiatric diagnosis, and those receiving short-term (2–4 months) rather than long-term (> 6 months) inpatient SUD treatment were at particular risk of relapse following inpatient treatment [24]. In the present study, many patients met at least one of these risk factors, which might be a reason for our findings. Another factor may be that inpatient detoxifications primarily aimed to stabilize patients and improve follow-up care without reducing substance use. If this is the case, the treatment was listed under the wrong heading and should instead be classified as a form of stabilization. Other outcome measures of their effectiveness may thus be needed.

Injecting substance users had a higher rate of substance use than those who did not inject at baseline and over time. According to previous studies [30, 31], it is also likely that high substance use predicts injecting substance use in this population. Injecting substances are associated with dependence and many health challenges [4, 32], resulting in overdoses and hospital admissions [32]. The broad range of comorbidities and complexities among injecting substance users emphasizes the need for coordinated medical and psychosocial SUD treatment to reduce substance use, in line with the European Union’s Drug Strategy for 2021–2025 [5]. Thus, various treatment approaches may be important for helping patients to recover from injecting substance use and managing their complex medical and psychosocial comorbidities [33].

Age over 60 years was associated with lower substance use than those under 30 years of age at baseline and over time. This points toward previous reports indicating lower substance use among older than younger SUD patients [2, 34]. Older SUD patients usually have more substance-related physical and mental comorbidities than younger patients, placing a higher responsibility on existing health care services. Previous observational studies have shown that receiving health services and being older were associated with more legal prescriptions for addictive medications compared with being younger, potentially making illegal substance use less likely [35–37]. This might explain the lower illegal substance use among older SUD patients. Moreover, in 2017, 59% of premature deaths from substance overdose were among individuals younger than 50 years globally [38], which suggests that individuals engaging in the most extensive substance use die before reaching 50. This might support our results by indicating that older SUD patients usually have lower illegal substance use than younger SUD patients.

## Strengths and limitations

This study has some strengths and limitations. We have included 708 SUD patients who are typically difficult to reach in health-care. Of those, 171 patients had follow-up measurements at least 1 year, making longitudinal analyses possible. However, these results should be interpreted cautiously because they only represented one out of four recruited patients. Three out of four patients were mainly recruited from OAT outpatient clinics, which could affect the generalizability of our results to other SUD populations. Moreover, due to clinical challenges, including systematic and patient delays, the health assessments were conducted at varying time intervals. This may complicate the interpretation of the predicted substance use changes from baseline. Moreover, the duration of inpatient detoxification and the time intervals between substance use measurements and inpatient detoxification were not considered in the analyses, which may reduce the results’ generalizability. Furthermore, the substance use changes were only estimated for patients who underwent outpatient SUD treatment throughout the study period, which means that the impact of entering SUD treatment on substance use was not considered. Moreover, more frequent substance use measurements could have identified possible fluctuations within shorter time intervals that might not necessarily be prolonged. Even so, our estimates are likely to have captured the general patterns.

## Conclusion

Inpatient detoxification was not associated with changes in substance use among patients receiving outpatient SUD treatment. Otherwise, injecting substance use was a particular risk factor for a high level of substance use. Reducing substance use is one of many goals of SUD treatment. Future research needs to evaluate the impact of other treatment approaches on substance use, ideally in randomized controlled trials.

## Supplementary Information

**Additional file 1.** The Substance Use Severity Index (SUSI) calculation. The calculation of SUSI based on the substance use during the past 12 months.

**Additional file 2 **Adjusted linear mixed model for the Substance Use Severity Index (SUSI) for patients with two or more SUSI measurements (*N* = 170). CI: Confidence Interval; OAT: Opioid Agonist Therapy; cSUSI: Mean Change in Substance Use Severity Index; SUD: Substance Use Disorder. ^1)^ The SUSI is a continuous variable ranging from 0 to 1, where 0 indicates no substance use and 1 indicates daily substance use for all substances (cannabis, amphetamines, cocaine, opioids, benzodiazepines, and alcohol). ^2)^ The value shows the difference in adjusted SUSI between “going to inpatient detoxification” and “not going to inpatient detoxification” at baseline (before detoxification). ^3)^ Interpretation: the cSUSI per year from baseline of a constant predictor and its comparator (e.g., the cSUSI of “ongoing injecting substance use” (constant predictor) and “no ongoing inject substance use” (constant comparator) per year from baseline). ^4)^ Interpretation: the cSUSI of a time-varying predictor and its time-varying comparator per year from baseline (e.g., the cSUSI per year of “going to inpatient detoxification” and “not going to inpatient detoxification” from baseline). The table displays a linear mixed model analysis (Restricted Maximum Likelihood regression) evaluating the impact of inpatient intoxication, injecting substance use, age, and gender on the SUSI at baseline and from baseline (over time) among patients undergoing outpatient SUD treatment with two or more substance use measurements.

## Data Availability

No additional data are available due to data protection requirements.

## Abbreviations

CI: Confidence interval
cSUSI: Mean change in substance use severity index
HCV: Hepatitis C virus
HIV: Human immunodeficiency virus
IQR: Interquartile range
OAT: Opioid agonist therapy
SD: Standard deviation
SUD: Substance use disorder
SUSI: Substance use severity index
