# Supplementary material for: Changes in substance use during outpatient treatment for substance use disorders: a prospective Norwegian cohort study from 2016 to 2020
Source: Subst Abuse Treat Prev Policy. 2021 Sep 15;16:67. doi: 10.1186/s13011-021-00403-9 (PMC8442290; doi:10.1186/s13011-021-00403-9)
Supplement: Supplementary file 1 — Additional file 1. The Substance Use Severity Index (SUSI) calculation. The calculation of SUSI based on the substance use during the past 12 months. [file 13011_2021_403_MOESM1_ESM.docx]

**Additional File 1**

| Substances use during the past 12 months | | | | | | |
| --- | --- | --- | --- | --- | --- | --- |
|  | Never | < 1 day per month | 1-3 days per month | 1-3 days per week | > 3 days per week | Daily |
|  | Score from zero to five | | | | | |
| Alcohol | 0 | 1 | 2 | 3 | 4 | 5 |
| Benzodiazepines | 0 | 1 | 2 | 3 | 4 | 5 |
| Cannabis | 0 | 1 | 2 | 3 | 4 | 5 |
| Opioids | 0 | 1 | 2 | 3 | 4 | 5 |
| Stimulants* | 0 | 1 | 2 | 3 | 4 | 5 |

*Stimulants include amphetamines and cocaine.

$$SUSI=\frac{Alcohol+Benzodiazepines+Cannabis+Opioids+Stimulants}{25}$$

SUSI: Substance Use Severity Index
