# Supplementary material for: Changes in substance use during outpatient treatment for substance use disorders: a prospective Norwegian cohort study from 2016 to 2020
Source: Subst Abuse Treat Prev Policy. 2021 Sep 15;16:67. doi: 10.1186/s13011-021-00403-9 (PMC8442290; doi:10.1186/s13011-021-00403-9)
Supplement: Supplementary file 2 — Additional file 2 Adjusted linear mixed model for the Substance Use Severity Index (SUSI) for patients with two or more SUSI measurements (N = 170). CI: Confidence Interval; OAT: Opioid Agonist Therapy; cSUSI: Mean Change in Substance Use Severity Index; SUD: Substance Use Disorder. 1) The SUSI is a continuous variable ranging from 0 to 1, where 0 indicates no substance use and 1 indicates daily substance use for all substances (cannabis, amphetamines, cocaine, opioids, benzodiazepines, and alcohol). 2) The value shows the difference in adjusted SUSI between “going to inpatient detoxification” and “not going to inpatient detoxification” at baseline (before detoxification). 3) Interpretation: the cSUSI per year from baseline of a constant predictor and its comparator (e.g., the cSUSI of “ongoing injecting substance use” (constant predictor) and “no ongoing inject substance use” (constant comparator) per year from baseline). 4) Interpretation: the cSUSI of a time-varying predictor and its time-varying comparator per year from baseline (e.g., the cSUSI per year of “going to inpatient detoxification” and “not going to inpatient detoxification” from baseline). The table displays a linear mixed model analysis (Restricted Maximum Likelihood regression) evaluating the impact of inpatient intoxication, injecting substance use, age, and gender on the SUSI at baseline and from baseline (over time) among patients undergoing outpatient SUD treatment with two or more substance use measurements. [file 13011_2021_403_MOESM2_ESM.docx]

**Additional File 2:** Adjusted linear mixed model for the Substance Use Severity Index

(SUSI)^1)^ for patients with two or more SUSI measurements (N = 170)

|  | Effect estimates | |
| --- | --- | --- |
|  | Coefficients (95 % CI) | p-value |
| Substance Use Severity Index (β_0_) | 0.29 (0.19;0.39) | < 0.001 |
| Time trend | 0.03 (-0.06;0.12) | 0.502 |
| *Baseline* | | |
| Female | 0.02 (-0.04;0.07) | 0.564 |
| Years of age:  < 30  30-40  40-50  50-60  ≥ 60 | 0.00 (ref.)  -0.02 (-0.11;0.08)  -0.06 (-0.15;0.04)  -0.04 (-0.14;0.06)  -0.05 (-0.17;0.07) | 0.723  0.245  0.419  0.398 |
| Injecting substance use | 0.17 (0.12;0.21) | < 0.001 |
| Inpatient detoxification^2)^ | 0.02 (-0.04;0.08) | 0.470 |
| *Predictors remain constant from baseline^3)^* | | |
| Injecting substance use | 0.02 (-0.02;0.06) | 0.295 |
| Female | -0.04 (-0.08;-0.01) | 0.023 |
| Years of age:  < 30  30-40  40-50  50-60  ≥ 60 | 0.00 (ref.)  -0.07 (-0.17;0.02)  -0.03 (-0.12;0.06)  -0.05 (-0.14;0.04)  -0.06 (-0.16;0.05) | 0.107  0.486  0.261  0.295 |
| *Time-varying predictors from baseline^4)^* | | |
| Inpatient detoxification | 0.00 (-0.04;0.04) | 0.941 |
| Starting to inject substances | 0.11 (0.08;0.15) | < 0.001 |
